# Sovateltide promotes regeneration and reduces β‐amyloid burden in a transgenic mouse model of Alzheimer’s disease

**DOI:** 10.1002/alz.095704

**Published:** 2025-01-09

**Authors:** Seema Briyal, Kathryn Roy, Anil Gulati

**Affiliations:** ^1^ Midwestern University, Downers Grove, IL USA; ^2^ Pharmazz, Inc, Willowbrook, IL USA

## Abstract

**Background:**

Alzheimer’s disease (AD) is a devastating condition with no known effective treatment. We have demonstrated cerebral endothelin B receptors (ETBR) as a potential target to treat AD. However, the mechanism of action of sovateltide remains elusive. Therefore, we investigated its mechanism of action in neuronal regeneration in APP/PS1 transgenic mouse model.

**Method:**

APP/PS1 transgenic mice and C57BL/6 control mice were divided into two groups (vehicle and sovateltide). Sovateltide (5 µg/kg) was intravenously injected, three times at 2‐hour intervals on days 1, 3 and 6 of every month until study end points (3‐, 6‐ and 12‐months age). Control mice received an equal volume of saline. Separate sets of mice were sacrificed at the end of 3, 6 and 12 months and beta amyloid plague formation, mitochondrial dysfunction, neuro‐ and synapto‐genesis were assessed in the brain.

**Result:**

APP/PS1 transgenic mice showed significant (p<0.001) impairment in spatial memory with elevated levels of Aβ plaque along with decreased levels of neuro and synaptogenic markers, which reflected the adverse effect of disease progression. Treatment with sovateltide rescued learning and memory deficits and significantly (p<0.001) decreased Aβ plaque load compared to vehicle in 6 months and 12 months aged transgenic mice. Moreover, sovateltide attenuated neuronal loss in the hippocampus and enhanced neurogenesis in APP/PS1 mice as evident by increased expression of NeuroD1 (p<0.0001), DoubleCortin (p<0.0001) and NeuN (p<0.0001) along with an upregulation of synaptic proteins (synapsin‐1, synaptophysin, and postsynaptic density‐95) compared to vehicle at 6 and 12 months. Sovateltide treatment also decreased mitochondrial fission (Drp1; p<0.001), increased mitochondrial fusion (Mfn2; p<0.001) and mitochondrial cross‐sectional area (p<0.01). Additionally, caspase‐3 level was significantly (p<0.001) higher in vehicle group than that in the sham group, and treatment with sovateltide significantly (p<0.01) reduced caspase‐3 level. There were no changes in the expression of any of these markers at three months.

**Conclusion:**

These findings suggest that sovateltide treatment promotes regeneration while simultaneously decreasing the pathology associated with Alzheimer’s disease.

